# Demand Response Strategy Considering Industrial Loads and Energy Storage with High Proportion of Wind-Power Integration

**DOI:** 10.3390/s24227335

**Published:** 2024-11-17

**Authors:** Chongyi Tian, Julin Li, Chunyu Wang, Longlong Lin, Yi Yan

**Affiliations:** Shandong Key Laboratory of Smart Buildings and Energy Efficiency, School of Information and Electrical Engineering, Shandong Jianzhu University, Jinan 250101, China

**Keywords:** wind-power consumption, industrial loads, hybrid energy storage, multiple time scales, energy management

## Abstract

To address the challenges of reduced grid stability and wind curtailment caused by high penetration of wind energy, this paper proposes a demand response strategy that considers industrial loads and energy storage under high wind-power integration. Firstly, the adjustable characteristics of controllable resources in the power system are analyzed, and a demand response scheduling framework based on energy storage systems and industrial loads is established. Building on this foundation, a multi-scenario stochastic programming approach is employed to develop a day-ahead and intra-day multi-time-scale optimization scheduling model, aimed at maximizing economic benefits. In response to the challenges of wind-power fluctuations with high temporal resolution, a strategy for smoothing intra-day wind-power variability is further proposed. Finally, simulations are conducted to derive optimal demand response strategies for different stages. As verified by the comparison of different scheduling strategies, the demand response strategy proposed in this paper can reduce wind curtailment when there is sufficient wind power and reduce load shedding when there is insufficient wind power, which effectively reduces the system operation cost.

## 1. Introduction

Wind energy, as a significant renewable resource, has experienced rapid global development in recent years [1]. Compared to traditional fossil fuel power generation, wind energy offers significant advantages in being clean, environmentally friendly, and renewable [2]. However, the intermittent and fluctuating characteristics of wind power make it face many challenges in the process of grid integration and consumption [3]. The output power of wind turbines is highly influenced by wind-speed fluctuations, leading to instability in generation and difficulties in achieving a good match with grid load. This imbalance between supply and demand causes wind power, in some periods of time, to show an “abandoned wind” phenomenon [4]; that is, although there are enough wind resources, due to the low load of the grid or scheduling inflexibility, part of the wind power cannot be connected to the grid for utilization, seriously restricting the further development of the wind-power industry [5]. Therefore, increasing the flexibility of the grid load is crucial to reduce wind curtailment [6].

With the integration of a high proportion of wind energy, the basic peak-shaving capacity of conventional thermal power units is insufficient to fully absorb wind power [7]. Therefore, it is necessary to incorporate more demand-side resources into the power system [8,9]. The literature [10] incorporates the demand response of electric vehicle customers, commercial customers, industrial customers, and residential customers into the wind-power absorption model to enhance wind-power utilization. The literature [11] considers the impact of users’ electricity consumption habits on grid peak-shaving, adds a price-responsive model, and constructs a wind–solar–thermal storage model, which reduces wind and light curtailment and improves the economics of power system peaking. The literature also guides users’ electricity consumption behavior through time-sharing tariffs and interruptible load management to alleviate the peak demand of the grid due to the intermittency of wind-power generation. The aforementioned studies effectively improve wind-power absorption during peak periods by integrating adjustable resources on the load side. However, these studies primarily focus on the demand response of residential and electric vehicle loads, which often have the disadvantages of slower response times, complex management, and technical and equipment limitations [12]. In contrast, industrial loads, characterized by high electricity consumption, concentrated equipment, high levels of automation, and fast response times, represent an excellent demand response resource [13]. Therefore, incorporating industrial loads into demand response offers a novel approach to enhance wind-power utilization and ensure the economic stability of the power system.

Current research has proposed various strategies for load regulation and demand response. The literature [14] analyzes physical processes to categorize the primary flexible loads in industrial parks into three types: high-energy-consuming industrial rotating loads, high-energy-consuming industrial heating loads, and energy storage loads. An intelligent offline database is then constructed to facilitate demand response. The literature [15] presents a novel multi-objective coordinated scheduling method for wind-power utilization, taking into account an energy-intensive load adjustment model to ensure the benefits of both the power system and energy-intensive loads. The literature [16] proposes a low-carbon scheduling method for the power system, considering the energy-intensive load of magnesium smelting to mitigate fluctuations in wind-power generation. This method integrates the strictly adjustable magnesium-smelting load with thermal-power plants on the demand side to optimize grid operations and collective scheduling. The literature [17] selects cement and aluminum-smelting plants as two typical industrial demand response resources (IDRR) to enhance wind-power penetration and achieve low-carbon benefits. The literature [18] presents a method using an energy-intensive chemical plant as a case study to evaluate the flexibility of power plants in providing electricity reserves while ensuring that production demands are met. The literature [19] proposes a synergistic optimization method that combines the regulation of electrolytic aluminum load (EAL) with deep peak-shaving of thermal power (DPS), effectively minimizing social peak-shaving costs and improving the operational economy of deep peak-shaving in thermal-power generation. The literature [20] indicates that, under conditions of high wind-power penetration and limited regulation capacity of conventional thermal-power plants, incorporating high-energy-consuming loads, such as electric arc furnaces, into system optimization scheduling not only enhances the capacity for wind-power absorption, but also effectively reduces carbon dioxide emissions.

Current research has extensively explored the participation of high-energy-consuming loads as demand response resources in grid scheduling to reduce wind and solar curtailment and lower carbon dioxide emissions. However, applied research on the co-scheduling of electrolytic aluminum load and electric arc furnace load in grid scheduling, especially in the field of demand response, is still insufficient. Further in-depth exploration and practical validation of these applications are urgently needed. Eastern China is rich in wind energy resources and has become an important base for wind-power generation both in China and globally. Despite the positive development trend of the wind-power industry, it still faces challenges such as market development and insufficient absorption of wind power. To address these issues, the auxiliary power service system has incorporated various adjustable loads into its scheduling framework, including traditional high-load industrial loads, interruptible loads from commercial and industrial sectors, and electric vehicle charging networks. These loads can respond to electricity scheduling commands, enhancing the power system’s regulatory capacity. In addition, Eastern China, as a hub for the electrolytic aluminum industry and steel industry, has abundant adjustable resources that provide strong support for wind-power absorption in the region [21,22]. In recent years, due to the intensification of market competition and the impact of the general environment, the industry has had overcapacity and increasing costs, and profit margins are gradually shrinking. On the one hand, enterprises can achieve additional economic benefits by participating in demand response mechanisms. On the other hand, electrolytic aluminum load and electric arc furnace load on the demand side, as adjustable load resources, can effectively collaborate with thermal-power plants. This cooperation would enhance the urban grid’s capacity to absorb renewable energy sources such as wind power. Additionally, it can alleviate the pressure on thermal-power plants during peak electricity demand periods, significantly reduce carbon emissions, and promote the development of a low-carbon economy. Therefore, making full use of these load resources in grid scheduling helps achieve efficient utilization of renewable energy and promotes the optimization and upgrading of the energy structure.

Additionally, the integration of energy storage systems is one of the key technologies to ensure the stable operation of power systems with a high penetration of wind power. Through energy storage devices, the power system can store excess electricity when wind-power generation exceeds demand and release this stored energy during peak load periods, thereby effectively reducing wind curtailment and improving the utilization efficiency of wind power [23,24]. Energy storage systems can dynamically balance electricity supply and demand through real-time charging and discharging, enhancing the grid’s adaptability to renewable energy sources like wind power and improving its flexibility and reliability [25,26,27]. In this context, deploying energy storage devices at wind farms is considered an effective way to reduce wind curtailment. However, due to the high costs of large-capacity battery storage systems, directly connecting wind power to the grid for on-site consumption is more practical and economical. Although battery storage systems do not directly absorb wind power, they can be used to mitigate the impact of wind-power fluctuations on the grid. The literature [28] proposes a rolling optimization strategy for microgrids that considers grid-connected power fluctuations, describing a microgrid model that includes a hybrid energy storage system with supercapacitors and batteries. Through a wind–storage combined generation system, the wind-power energy storage system can conduct energy scheduling and management on a finer time scale. This optimized scheduling dynamically adjusts electricity supply based on real-time wind-power output, storage status, and grid demand, reducing the impact of wind-power fluctuations on the grid.

Based on the above research background, this paper proposes a multi-time-scale wind-power consumption strategy considering industrial loads and energy storage. A scheduling framework based on energy storage and industrial loads is developed, with a detailed analysis of their regulation characteristics. A multi-time-scale scheduling framework is designed, integrating energy storage and various industrial loads. A multi-scenario stochastic programming method is employed to establish a multi-time-scale scheduling model with economic optimization as the objective. Finally, the effectiveness of the proposed strategy is validated through case studies.

## 2. Demand Response Scheduling Framework Based on Energy Storage and Industrial Loads

### 2.1. Analysis of Load Characteristics of Electrolytic Aluminum

#### 2.1.1. Characteristics of Electrolytic Aluminum Load Operation

**Figure 1:**
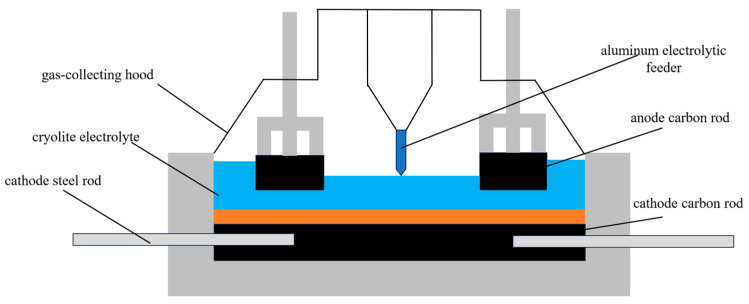
Electrolytic aluminum production process.

Electrolytic aluminum is the primary method for refining aluminum in modern industry, usually using the cryolite-alumina molten salt electrolysis process. Using alumina as the raw material, the process takes place in an electrolytic cell, which uses carbonaceous material as the cathode and anode and passes hundreds of kiloamperes of direct current between the two electrodes for electrolysis. The energy consumption of the process is also very high due to the extremely high current intensity of the electrolytic cell. The electrolytic aluminum production process is shown in Figure 1.

As a typical high-energy-consuming load, the cost of electricity in the production of electrolytic aluminum usually accounts for 30–40% of the total production cost, and companies are strongly willing to participate in demand response. The electrolytic aluminum process has a long duration and a relatively stable load, so it can be regarded as a constant power load with a power curve close to the horizontal line, which is convenient for load forecasting. At the same time, electrolytic aluminum has thermal energy storage characteristics, the thermal inertia time constant is large, and a short period of time to adjust the load of electrolytic aluminum will not significantly affect the production. In addition, the electrolytic aluminum production line has a high degree of automation and can accept dispatch instructions to adjust production. Therefore, the electrolytic aluminum load has a certain degree of adjustability and can be used as a regulating load in the power demand response, which can help to cut peaks and fill valleys and reduce the cost of enterprise production cost.

Due to the precision of electrolytic components in electrolytic aluminum production, the load should not be adjusted frequently, and a period of stable operation is required after power adjustments. Therefore, the aluminum electrolysis load is more suitable as a day-ahead scheduling resource for load optimization, allowing effective power system regulation while meeting process requirements.

#### 2.1.2. Electrolytic Aluminum Load Scheduling Model

The active power of electrolytic aluminum is shown in Equation (1).

(1) $$P_{t}^{LV}=N_{LV}P_{base}^{LV}+\sum_{n=1}^{N_{LV}}\mu_{n,t}^{LV}\Delta P_{n}^{LV}$$

where $P_{t}^{LV}$ is the total power of electrolytic aluminum; $N_{LV}$ is the number of electrolytic cell; $P_{base}^{LV}$ is the average power of electrolytic aluminum; $\mu_{n,t}^{LV}$ is a binary variable, where $\mu_{n,t}^{LV}$ is 1 when the load of the electrolytic aluminum is adjusted, and vice versa is 0; $\Delta P_{n}^{LV}$ is the adjustable power of the aluminum electrolysis load for the *n*th electrolytic cell.

The regulation power constraint is shown in Equation (2).

(2) $$\Delta P_{n}^{LV\min}\leq \Delta P_{n}^{LV}\leq \Delta P_{n}^{LV\max}$$

where $\Delta P_{n}^{LV\max}$ and $\Delta P_{n}^{LV\min}$ are the upper and lower limits of the adjusted power of the *n*th electrolytic cell, respectively.

The regulation number constraint is shown in Equation (3).

(3) $$0\leq \sum_{t=2}^{T}\left|\mu_{n,t}^{LV}-\mu_{n,t-1}^{LV}\right|\leq M$$

where *M* is the maximum number of adjustments.

Since thermal balance has to be ensured in the process of electrolytic aluminum generation, it is required that the load increase and decrease in capacity are equal during the system scheduling cycle.

(4) $$\sum_{t=1}^{N_{T}}\Delta P_{n}^{LV}=0$$

### 2.2. Analysis of Electric Arc Furnace Load Characteristics

#### 2.2.1. Characteristics of Electric Arc Furnace Load Operation

The electric arc furnace (EAF) is commonly used in the steel industry, primarily for steel smelting through arc heating. During production, after the furnace is loaded, the EAF first lowers the electrodes to contact the furnace material, then begins heating by supplying electricity. An electric arc forms between the electrodes and the furnace material, heating the furnace material to its melting temperature. The arc generates a large amount of heat during this process, causing the metal to melt gradually. During the melting process, the position of the electrode, current, voltage, etc., can be adjusted according to the temperature requirements and the power load of the arc furnace to ensure the stability of the arc and power output. The factory can regulate the arc furnace load to more constant voltage through using an electrode-lifting device to adjust the position of the electrode to change the amplitude of the three-phase current, so as to accurately regulate the arc furnace load. Modern electric arc furnace equipment is usually equipped with an intelligent control system and grid scheduling interface, which can monitor the various operating parameters of the electric arc furnace in real time and feed the information back to the grid scheduling system. Through this real-time information interaction, the power grid can flexibly dispatch the load of the electric arc furnace according to the actual demand, so that it can increase or decrease the power output in the appropriate time period to realize the optimal allocation of power resources.

Electric arc furnaces, as typical equipment with a high-energy-consumption load, consume a large amount of electrical energy in the course of use. They are equipped with a control system that has a fast response characteristic and can flexibly adjust the power output. At the same time, in order to respond to changes in product demand, the factory will continue to optimize the production plan, so that the load of the electric arc furnace can be dynamically adjusted within a wide range. As a result, electric arc furnace loads are characterized by greater regulation potential, rapid responsiveness, and continuous adjustment, and have the significant advantage of being an intra-day demand response resource.

#### 2.2.2. Electric Arc Furnace Scheduling Model

The active power of the electric arc furnace type load is shown in Equation (5)

(5) $$P_{n,t}^{EAF}=\sqrt{3}U_{n,t}^{EAF}I_{n,t}^{EAF}\cos\varphi$$

where $P_{n,t}^{EAF}$ is the power of the *n*th electric arc furnace at time *t*; $U_{n,t}^{EAF}$ and $I_{n,t}^{EAF}$ are the voltage and current of the *n*th electric arc furnace at time *t*. cosφ is the power factor of the power supply system.

Load regulation of electric arc furnaces has to be carried out in a way that ensures production quality and safety, and therefore has the following constraints.

(6) $$P_{t}^{EAF}=N_{EAF}P_{\;base}^{EAF}+\sum_{n=1}^{N_{EAF}}\mu_{n,t}^{EAF}\Delta P_{n}^{EAF}$$

(7) $$\Delta P_{n}^{EAF\min}\leq \Delta P_{n,t}^{EAF}\leq \Delta P_{n}^{EAF\max}$$

where $P_{t}^{EAF}$ is the total electric arc furnace power; $N_{EAF}$ is the number of electric arc furnaces; $P_{\;base}^{EAF}$ is the average power of the electric arc furnace; $\mu_{n,t}^{EAF}$ is a binary variable, 1 when the electric arc furnace load is adjusted, and vice versa; $\Delta P_{n}^{EAF}$ is the adjustable power of the electric arc furnace; $\Delta P_{n}^{EAF\max}$ and $\Delta P_{n}^{EAF\min}$ are the upper and lower limits of the adjusted power of the *n*th electric arc furnace, respectively.

### 2.3. Pumped Storage Power Station Characteristics Analysis

Pumped storage consists of reversible turbines, an upper reservoir, and a lower reservoir. Compared to the uncertainty of wind and solar power output, hydropower units, including pumped storage, possess strong controllability and excellent regulation capabilities. However, they have low energy density, slow charge and discharge response speeds, and are subject to geographical limitations [29,30,31]. Eastern China has abundant water resources and a large number of small- and medium-sized pumped storage power plants. Therefore, in this paper, pumped storage is considered as a day-ahead demand response resource.

### 2.4. Hybrid Battery Energy Storage System Characteristics Analysis

Hybrid energy storage systems usually combine different types of energy storage technologies, such as batteries and supercapacitors, flywheels, and batteries. Each of the different energy storage technologies has unique charging and discharging characteristics and response rates [32]. Through optimized combinations, these storage technologies can work together on different time scales to achieve smoother wind-power consumption. For example, supercapacitors are able to respond quickly to short-term wind-power fluctuations, while batteries are suited to handle energy storage and release on longer time scales, resulting in an overall increase in wind-power consumption [33]. Hybrid energy storage systems are able to flexibly regulate the operation of energy storage devices under different wind-power output conditions. In case of large fluctuations in wind-power output, the hybrid energy storage system can be quickly adjusted to maintain grid stability.

## 3. Multi-Time-Scale Framework for Scheduling Involving Storage and Various Industrial Loads

The day-ahead scheduling plan optimizes the time periods for the next 24 h, with a resolution of 1 h. Based on the forecasted load and wind-power data, a coordinated scheduling model is developed for thermal-power units, pumped storage, and electrolytic aluminum load. The start–stop plan of thermal-power units, the scheduling plan of electrolytic aluminum load, and the charging and discharging volumes of pumped storage are used as fixed conditions to be incorporated into the intra-day scheduling process.

**Figure 2:**
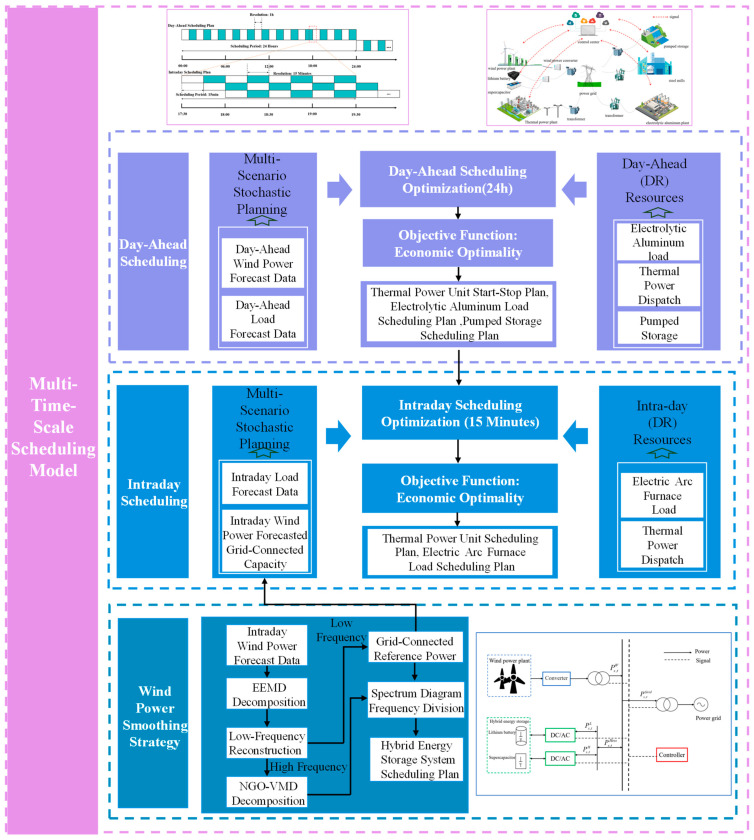
Multi-time-scale optimization scheduling framework.

The intra-day rolling optimization focuses on the next 4-h period, with a resolution of 15 min. Based on intra-day forecast information, electric arc furnaces enterprises are incorporated into the model to adjust the day-ahead scheduling plan and correct deviations. The charging and discharging volumes of battery energy storage, the power generation of thermal-power units, and the scheduling plan for electric arc furnace load are specified in this process. The framework diagram for multi-time-scale scheduling is shown in Figure 2.

### 3.1. Day-Ahead Scheduling Strategy

In the day-ahead stage, a multi-scenario stochastic programming method is employed. This method can fully account for future uncertainties by planning across multiple potential scenarios, thereby enhancing the robustness and flexibility of decision-making. The day-ahead demand response resources include electrolytic aluminum load, thermal-power units, and pumped storage power stations.

#### 3.1.1. Objective Function

(8) $$\min f_{daybefore}=\sum_{t=1}^{T}\left(f_{t}^{G}+f_{t}^{Es}+f_{t}^{Cut}+f_{t}^{DR}\right)$$

(9) $$\left\{\begin{array}{l} f_{t}^{G}=\sum_{s=1}^{N_{s}}\sum_{g=1}^{G}\left\{P_{s}\left[a_{g}{\left(P_{g,s,t}^{G}\right)}^{2}+b_{g}P_{g,s,t}^{G}+c_{g}\right]+Q_{g}(1-u_{g,s,t-1}^{G})u_{g,s,t}^{G}\right\}\\ f_{t}^{Es}=\sum_{s=1}^{N_{s}}P_{s}(P_{s,t}^{Water}C(P_{s,t}^{Water}))\\ f_{t}^{Cut}=\sum_{s=1}^{N_{s}}P_{s}(k^{Wcut}P_{s,t}^{Wcut}+k^{Lcut}P_{s,t}^{Lcut})\\ f_{t}^{DR}=(k_{a}^{DRA+}P_{t}^{DRA+}+k_{a}^{DRA-}P_{t}^{DRA-}) \end{array}\right.$$

where $f_{daybefore}$ represents the total operating cost of the system. $f_{t}^{G}$, $f_{t}^{Es}$, $f_{t}^{Cut}$ and $f_{t}^{DR}$ represent the costs of thermal-power units, pumped storage, wind curtailment, and demand response load, respectively. *N_s_* represents the number of scenarios; *G* represents the number of thermal-power units; *P_s_* is the probability of occurrence of scenario *s*; *a_g_*, *b_g_*, *c_g_* are the cost coefficients for the thermal-power units; *Q_g_* is the startup cost coefficient for the thermal-power units; $u_{g,s,t}^{G}$ represents the operating state of unit *g* at time *t* under scenario *s*; $P_{s,t}^{Water}$ represents the pumping and discharging power of the pumped storage at time *t* under scenario *s*; $C(P_{s,t}^{Water})$ represents the cost function for the pumped storage system; $P_{t}^{DRA+}$ and $P_{t}^{DRA-}$ represent the increased and decreased loads at the electrolytic aluminum load at time *t*, respectively. $k_{a}^{DRA+}$ and $k_{a}^{DRA-}$ represent the compensation costs for the increased and decreased electrolytic aluminum load, respectively; $P_{s,t}^{Wcut}$ and $P_{s,t}^{Lcut}$ represent the wind-power curtailment and load shedding at time *t* under scenario *s*, respectively; *k^Wcut^* and *k^Lcut^* represent the costs associated with lost load and wind-power curtailment, respectively.

#### 3.1.2. Constraints

(1) Power balance constraint

(10) $$P_{s,t}^{W}+\sum_{g=1}^{G}P_{g,s,t}^{G}+P_{s,t}^{Water}-P_{s,t}^{Wcut}=P_{s,t}^{L}+P_{t}^{DRA}-P_{s,t}^{Lcut}\;$$

where $P_{s,t}^{W}$ represents the wind-power output at time *t* under scenario *s*; $P_{s,t}^{L}$ represents the system load at time *t* under scenario *s*.

(2) Electrolytic aluminum constraints

The electrolytic aluminum production constraints are satisfied by Equations (1)–(4).

(3) Thermal-power units constraints

The constraints for the thermal-power units include output constraints and ramping constraints.

(11) $$u_{g,s,t}^{G}P_{g}^{\min}\leq P_{g,s,t}\leq u_{g,s,t}^{G}P_{g}^{\max}$$

(12) $$-\Delta U_{g}^{G}\leq P_{g,s,t}^{G}-P_{g,s,t-1}^{G}\leq \Delta U_{g}^{G}$$

where $u_{g,s,t}^{G}$ is a binary variable representing the on/off state of thermal generator *g* at time *t* under scenario *s*, where 1 indicates that the generator is on, and 0 indicates that it is off. $P_{g}^{\min}$ and $P_{g}^{\max}$ represent the minimum and maximum output power of thermal generator *g*, respectively. $\Delta U_{g}^{G}$ is the maximum load adjustment rate for thermal generator *g* at each time step.

(4) Pumped storage constraints

The constraints of pumped storage power station are mainly the operating power constraint of the reservoir, the water capacity constraint, and the rate of climb constraint.

(13) $$P_{water}^{\min}\leq P_{s,t}^{Water}\leq P_{water}^{\max}$$

(14) $$V_{Pump}^{\min}\leq V_{water}\leq V_{Pump}^{\max}$$

(15) $$-\Delta P_{Pump}\leq |P_{s,t}^{Water}-P_{s,t-1}^{Water}|\leq \Delta P_{Pump}$$

where $P_{water}^{\max}$ and $P_{water}^{\min}$ represent the upper and lower limits of the pumped storage power station’s grid connection power, respectively. $V_{Pump}^{\max}$ and $V_{Pump}^{\min}$ are the upper and lower limits of the water storage capacity of the pumped storage power station, respectively. $\Delta P_{Pump}$ is the ramp rate of the pumped storage power station.

(5) Wind-power constraint

The consumption of wind power must be less than the predicted value, satisfying the following constraints.

(16) $$0\leq P_{s,t}^{W}\leq P_{s,t}^{fore}$$

where $P_{s,t}^{fore}$ represents the predicted wind-power output at time *t* under scenario *s*.

(6) Load shedding and wind curtailment constraints

Load shedding and wind curtailment must be below the forecast for the day and meet the following constraints.

(17) $$0\leq P_{s,t}^{Wcut}\leq P_{s,t}^{Wfore}$$

(18) $$0\leq P_{s,t}^{Lcut}\leq P_{s,t}^{Lfore}$$

where $P_{s,t}^{Wfore}$ and $P_{s,t}^{Lfore}$ represent the predicted wind-power output and system load, respectively.

### 3.2. Intra-Day Wind-Power Fluctuation Smoothing Strategy

First, the ensemble empirical mode decomposition (EEMD) algorithm is used to extract the low-frequency signals from the original wind-power data as the grid-connected expected power, while the high-frequency signals represent the fluctuation power that the energy storage system needs to smooth. Next, fuzzy entropy is selected as the fitness function, and the northern goshawk—variational mode decomposition (NGO-VMD)—optimizes decomposed energy storage system power. The marginal spectrum obtained from the Hilbert transform determines the appropriate boundary between high- and low-frequency components. The low-frequency components are absorbed by lithium battery, while the high-frequency components are handled by supercapacitor.

The principle of EEMD involves adding white noise perturbations to the original signal to be decomposed, followed by applying empirical mode decomposition (EMD) to the processed signal. This process is repeated multiple times, and the results are averaged. Since the EEMD method employs the techniques of adding noise and averaging, it can more accurately extract the intrinsic modes of the signal compared to the traditional EMD method. This reduces the occurrence of spurious components during the decomposition process, thereby enhancing the overall accuracy of the decomposition [34].

The results obtained from EEMD decomposition are used for low-frequency reconstruction. This involves starting with the intrinsic mode function (IMF) of the lowest frequency and gradually adding each order of low-frequency reconstruction components until the fluctuation of the reconstructed signal exceeds the set grid fluctuation threshold. This point is designated as the decomposition point for grid-connected power and the hybrid energy storage system. The low-frequency reconstruction process is shown in Equation (19).

(19) $$\left\{\begin{array}{l} c2f\left(1\right)=RES\\ c2f\left(2\right)=RES+IMFh\\ \vdots \\ c2f\left(k+1\right)=RES+IMFh+\cdots +IMF1 \end{array}\right.\;$$

where *c*2*f* denotes the low-frequency reconstruction, i.e., from the residual component *RES* gradually to the *IMF* components of each order, with low frequency continuously superimposed.

The core of the EEMD algorithm is to decompose wind-power signals using the EMD algorithm. However, repeatedly applying the EMD algorithm to the same signal can lead to excessive decomposition of certain modes, causing frequency mixing between modes and exacerbating endpoint effects. To address these issues, the VMD algorithm can be used for a secondary decomposition of the high-frequency components obtained. In the VMD algorithm, the number of modes *K* and the penalty factor *α* are usually selected based on empirical methods, which can introduce subjectivity and lead to either over-decomposition or under-decomposition. Therefore, this study employs the northern goshawk optimization algorithm to optimize the values of *K* and *α* to enhance the accuracy and stability of the decomposition results [35].

The northern goshawk optimization algorithm, as a novel intelligent optimization method, demonstrates excellent optimization performance and broad application prospects by simulating the hunting behavior of northern goshawks in nature.

In the first stage, after identifying its prey, the northern goshawk swiftly migrates toward the target and launches an attack. This process is executed in the search space by randomly locating the target, effectively enhancing the exploration capability of the algorithm. The behavior of the northern goshawk can be expressed as follows:

(20) $$P_{i}=X_{k}(i=1,2,\cdots N,k=1,2,\cdots j-1,i+1,\cdots N)$$

(21) $$X_{i,j}^{new,p_{1}}=\left\{\begin{array}{l} X_{i,j}+r(p_{i,j}-lx_{i,j}),F_{P_{i}}<F_{i}\\ X_{i,j}+r(x_{i,j}-p_{i,j}),F_{P_{i}}>F_{i} \end{array}\right.$$

(22) $$X_{i}=\left\{\begin{array}{l} X_{i}^{new,p_{i}},F_{i}^{new,p_{1}}<F_{i}\\ X_{i},\;\;\;\;\;\;\;F_{i}^{new,p_{1}}\geq F_{i} \end{array}\right.$$

where *P_i_* represents the position of the prey selected by the *i*-th goshawk; $F_{P_{i}}$ denotes the corresponding fitness value; *k* is a uniformly distributed random natural number; $X_{i,j}^{new,p_{1}}$ is the new position of the *i*-th northern goshawk in the *j*-th dimension during the first stage; $X_{i}^{new,p_{i}}$ is the new position of the *i*-th northern goshawk; *p_i,j_* represents the prey position of the *i*-th northern goshawk in the *j*-th dimension; $F_{i}^{new,p_{i}}$ represents the corresponding fitness value; r ∈ (0,1) is a uniformly distributed random number; *l* is a random number that can be either 1 or 2. *r* and *l* are used to generate a random northern goshawk behavior during the search and update process.

**Figure 3:**
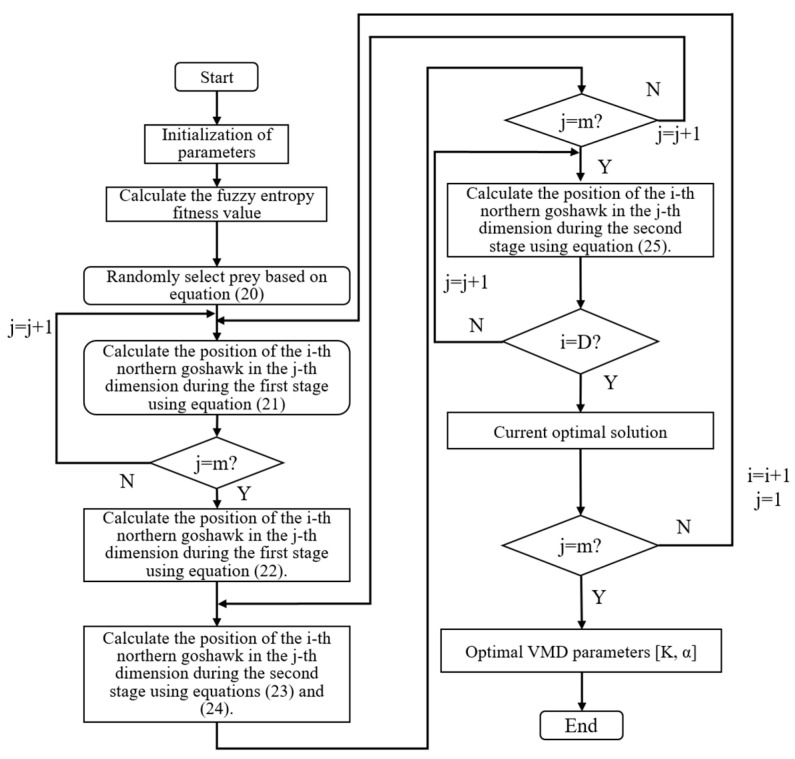
Flowchart for NGO optimization of VMD parameters.

In the second phase, it quickly chases the prey to complete the hunt. This mimicry of the chasing behavior enhances the algorithm’s local search capability. The behavior of the northern goshawk can be represented as follows:

(23) $$X_{i,j}^{new,p_{2}}=x_{i,j}+R(2r-1)x_{i,j}$$

(24) $$R=0.02(1-\frac{t}{T})$$

(25) $$X_{i}=\left\{\begin{array}{l} X_{i}^{new,P_{2}},F_{i}^{new,P_{2}}<F_{i}\\ X_{i},\;\;\;\;\;\;\;\;F_{i}^{new,P_{2}}\geq F_{i} \end{array}\right.$$

where *T* is the total number of iterations; *t* is the current iteration; *R* represents the chasing radius; $X_{i,j}^{new,P_{2}}$ is the new position of the *i*-th northern goshawk in the *j*-th dimension during the second phase; $F_{i}^{new,P_{2}}$ is the corresponding fitness value. The flowchart for the NGO algorithm optimizing the VMD parameters is shown in Figure 3.

Compared to distributed energy storage, centralized energy storage can more effectively conduct unified scheduling and control, optimizing the charging and discharging strategies of energy storage devices and improving overall system efficiency. Based on this, this paper employs centralized energy storage connected to the wind farm. The low-frequency grid-connected power after EEMD decomposition of the original wind power is denoted as $P_{s,t}^{Grid}$, while the portion of high-frequency disturbances absorbed by the hybrid energy storage system is denoted as $P_{s,t}^{Hess}$.

(26) $$P_{s,t}^{Hess}=P_{s,t}^{W}-P_{s,t}^{Grid}$$

**Figure 4:**
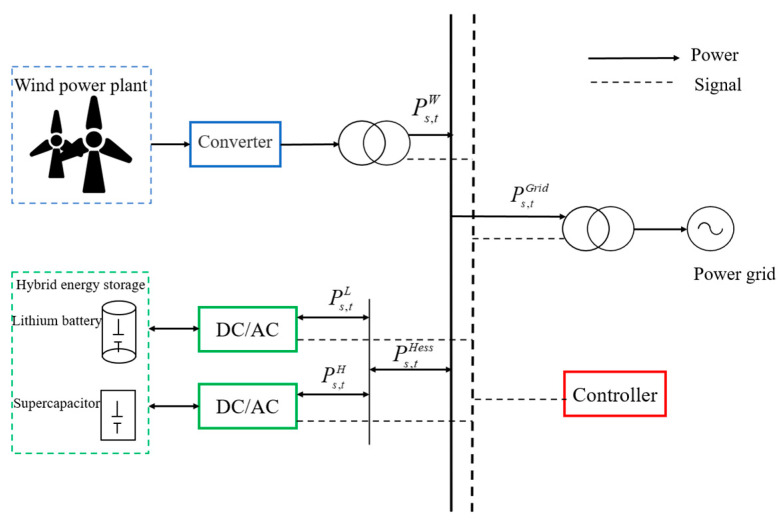
Structure diagram of the wind–hybrid storage system.

The fluctuation power frequency decomposition method based on the NGO-VMD algorithm will perform a secondary decomposition, breaking the fluctuation power into two parts: a smaller amplitude high-frequency component and a larger amplitude low-frequency component. The high-frequency component is smoothed by power-type energy storage devices, such as supercapacitors ($P_{s,t}^{H}$), while the low-frequency component is smoothed by energy-type energy storage devices, such as lithium batteries ($P_{s,t}^{L}$). This approach optimizes the operational efficiency of the hybrid energy storage system and stabilizes the output of wind power by reasonably allocating storage tasks. The structure of the wind federation storage system is shown in Figure 4.

(27) $$P_{s,t}^{Hess}=P_{s,t}^{L}+P_{s,t}^{H}$$

**Figure 5:**
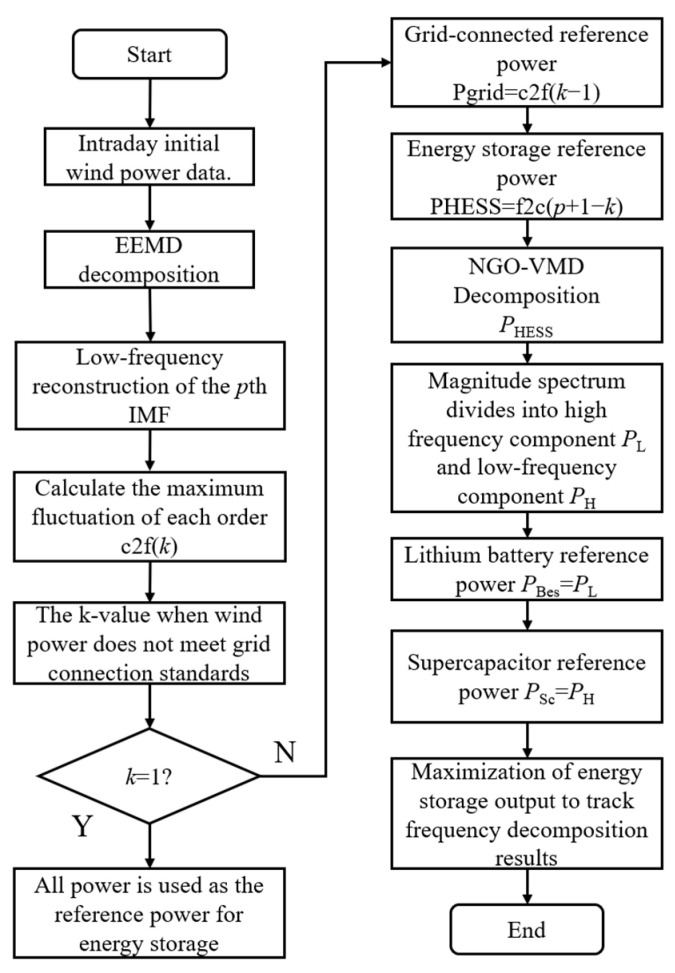
Intra-day wind-power fluctuation smoothing flowchart.

To ensure the wind-power smoothing effect of the hybrid energy storage system, the objective function is set to maximize the output of the energy storage while tracking the frequency decomposition results. The process of smoothing out wind-power fluctuations is shown in Figure 5.

(28) $$\min\left|P_{s,t}^{Bes}-P_{s,t}^{L}\right|$$

(29) $$\min\left|P_{s,t}^{Sc}-P_{s,t}^{H}\right|$$

(1) Power constraints for both types of energy storage during their operating cycles.

(30) $$-P_{\max}^{bes}\leq P_{s,t}^{Bes}\leq P_{\max}^{bes}$$

(31) $$-P_{\max}^{Sc}\leq P_{s,t}^{Sc}\leq P_{\max}^{Sc}$$

where $P_{\max}^{bes}$ and $P_{\max}^{Sc}$ are the maximum power of charging and discharging of the lithium battery and supercapacitor.

(2) SOE represents the percentage of the remaining energy relative to the rated capacity of the battery, and is used to reflect the state of charge, as it intuitively shows the available energy of the battery, providing a basis for decision-making in power scheduling. Therefore, SOE is used to measure the stored energy of energy storage devices.

(32) $$SOE_{s,t+1}^{Bes}=SOE_{s,t}^{Bes}+\frac{P_{s,t}^{Bes}\times \eta_{Bes}\times \Delta t}{E_{Bes}}$$

(33) $$SOE_{s,t+1}^{Sc}=SOE_{s,t}^{Sc}+\frac{P_{s,t}^{Sc}\times \eta_{Sc}\times \Delta t}{E_{Sc}}$$

where $SOE_{s,t}^{Bes}$ and $SOE_{s,t}^{Sc}$ are the energy states of the lithium battery and supercapacitor at time *t* in scenario *s*; $\eta_{Bes}$ and $\eta_{Sc}$ are the charging and discharging efficiencies of lithium battery and supercapacitor, respectively; $E_{Bes}$ and $E_{Sc}$ are the capacity of lithium battery and supercapacitor, respectively

(34) $$SOE_{b\min}<SOE_{s,t}^{Bes}<SOE_{b\max}$$

(35) $$SOE_{s\min}<SOE_{s,t}^{Sc}<SOE_{s\max}$$

where $SOE_{b\min}$ and $SOE_{b\max}$ are the lower and upper limits of the energy stored in the lithium battery, respectively. $SOE_{s\min}$ and $SOE_{s\max}$ are the lower and upper limits of the energy storage state of the supercapacitor, respectively

### 3.3. Intra-Day Optimization Scheduling Model

#### 3.3.1. Objective Function

(36) $$\min f_{inday}=\frac{1}{4}\sum_{t=1}^{T}\left(f_{t}^{G}+f_{t}^{ES}+f_{t}^{DR}+f_{t}^{cut}\right)$$

(37) $$f_{t}^{DR}=\sum_{i=1}^{N_{b}}k^{DRB}P_{i,t}^{DRB}$$

where *N_b_* represents the number of electric arc furnaces participating in the demand response; $k^{DRB}$ represents the compensation price for the electric arc furnaces load’s participation in the demand response; $P_{i,t}^{DRB}$ is the power of the electric arc furnace load of the *i*th electric arc furnace at time *t* participating in the demand response. Since the intra-day time resolution is 15 min, the objective function must be multiplied by 1/4.

#### 3.3.2. Constraints

(1) Power balance constraint

(38) $$P_{s,t}^{Grid}+\sum_{i=1}^{N_{G}}P_{s,t}^{G}+P_{s,t}^{Water}-P_{s,t}^{Wcut}=P_{s,t}^{L}+P_{t}^{DRA}+\sum_{i=1}^{N_{b}}P_{i,t}^{DRB}-P_{s,t}^{Lcut}$$

(2) Electric arc furnace load constraints

The electric arc furnace load constraints are represented by Equations (5)–(7).

(3) Other constraints.

The constraints for thermal-power units, pumped storage, load shedding, and wind curtailment are the same as those in the day-ahead scheduling model and are not repeated here.

## 4. Example Analysis

**Figure 6:**
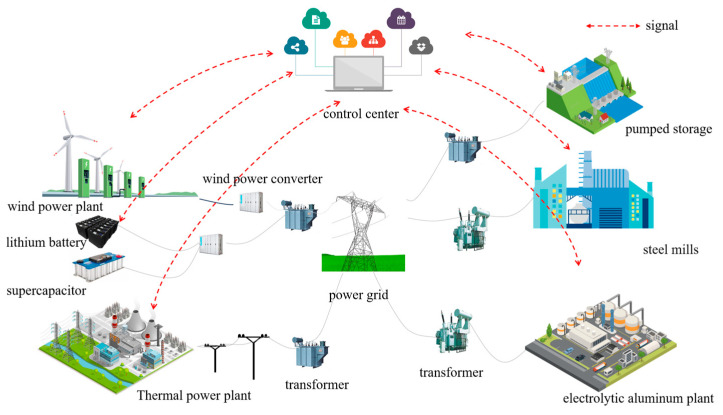
Power system diagram with various adjustable resources.

**Figure 7:**
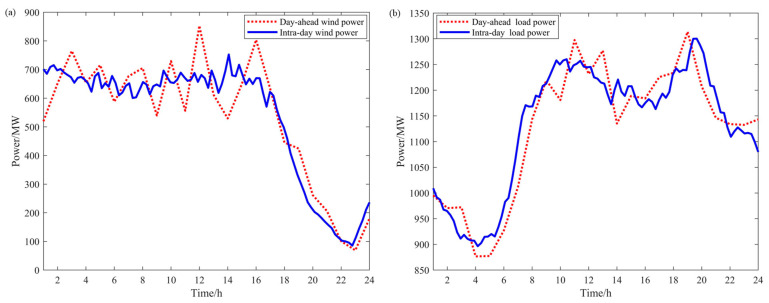
Wind-power and load forecast data in the sufficient wind-power scenario: (**a**) day-ahead and intra-day wind-power forecast data; (**b**) day-ahead and intra-day load forecast data.

**Figure 8:**
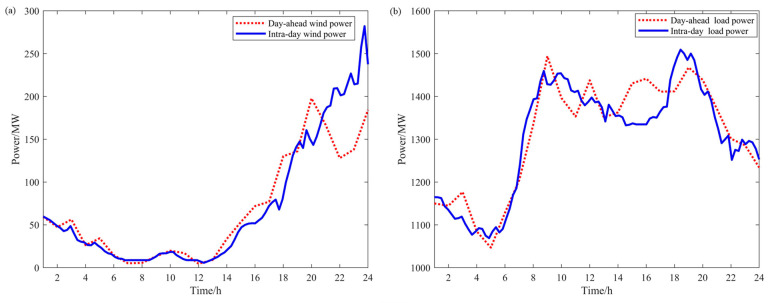
Wind-power and load forecast data in the insufficient wind-power scenario: (**a**) day-ahead and intra-day wind-power forecast data; (**b**) day-ahead and intra-day load forecast data.

In order to verify the validity of the method proposed in this paper, a simulation study is carried out in this paper. A power system containing multiple adjustable resources is contemplated, and the system schematic is shown in Figure 6. The data from a steelmaking factory and an electrolytic aluminum factory in China serve as the basis for the analysis in this paper. The electrolytic aluminum load is operated at a rated power of 307.2 MW, with an upward and downward capacity of 10% and 30% of the rated power, respectively. The compensation price is 60 CNY/WM·h. The electric arc furnace load is rated at 200 MW, its upward and downward adjustment capacities are 20% and 10% of the rated power, and the compensation price is 220 CNY/WM·h. The wind curtailment price is set at 600 CNY/WM·h. The load-shedding penalty cost is set at 4000 CNY/MW·h. The parameters of the thermal-power units in the system are shown in Table 1, and the parameters of the energy storage devices are shown in Table 2. The wind-power prediction data in the sufficient wind-power scenario are shown in Figure 7a; the load prediction data are shown in Figure 7b; the wind-power prediction data in the insufficient wind-power scenario are shown in Figure 8a; and the load prediction data are shown in Figure 8b.

### 4.1. Analysis of Day-Ahead Optimization Scheduling Results

**Figure 9:**
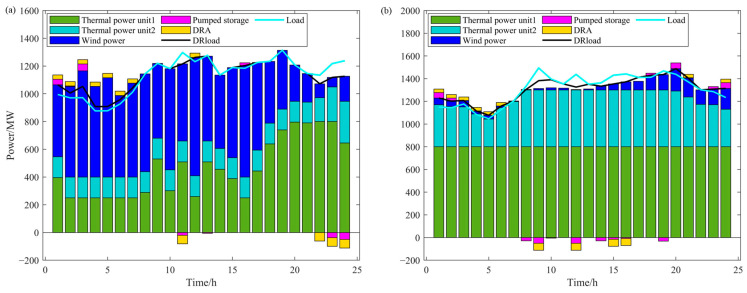
Day-ahead scheduling results: (**a**) day-ahead scheduling in the sufficient wind-power scenario; (**b**) day-ahead scheduling in the insufficient wind-power scenario.

From the wind-power and load forecast data in Figure 7 and the day-ahead scheduling results in the sufficient wind-power scenario in Figure 9a, it can be observed that under sufficient wind-power conditions, the increase in electrolytic aluminum load mainly occurs during periods 1–7 and period 12. During these periods, wind-power output is high while system load demand is low, especially in periods 2–6 and 12, where thermal-power units are already operating at minimum output and cannot reduce further to increase wind-power utilization. Therefore, in these periods, the only way to balance power supply and demand is by increasing the load to achieve a higher level of wind-power consumption. To ensure the stability of the electrolytic aluminum load throughout the scheduling period, part of the load is reduced and shifted to periods 11 and 22–24. The load level in period 11 is relatively high, so a moderate reduction in the electrolytic aluminum load will not significantly impact wind-power utilization. In periods 22–24, when wind-power output is lower and system load demand is higher, reducing the electrolytic aluminum load helps to alleviate the output pressure on thermal-power units, further optimizing the flexibility and economic efficiency of system operation.

From the wind-power and load forecast data in Figure 8 and the day-ahead scheduling results in the insufficient wind-power scenario in Figure 9b, it can be seen that under low wind-power conditions, the reduction in electrolytic aluminum load occurs at times 9, 12, 15, and 16. During these times, wind-power output is low while load demand is high, and thermal-power generation has already reached its maximum. Reducing the electrolytic aluminum load at these times helps decrease load-shedding costs. The increased electrolytic aluminum load is shifted to times 1–6, 21, and 24, when load demand is relatively low, and thermal-power generation still has room for adjustment to meet the increased load.

In addition, since thermal-power unit 1 is more economical than thermal-power unit 2, it is prioritized for adjustment. The charging and discharging of pumped storage follow a trend similar to the increase and decrease in electrolytic aluminum load.

### 4.2. Analysis of Intra-Day Wind-Power Fluctuation Smoothing Results

**Figure 10:**
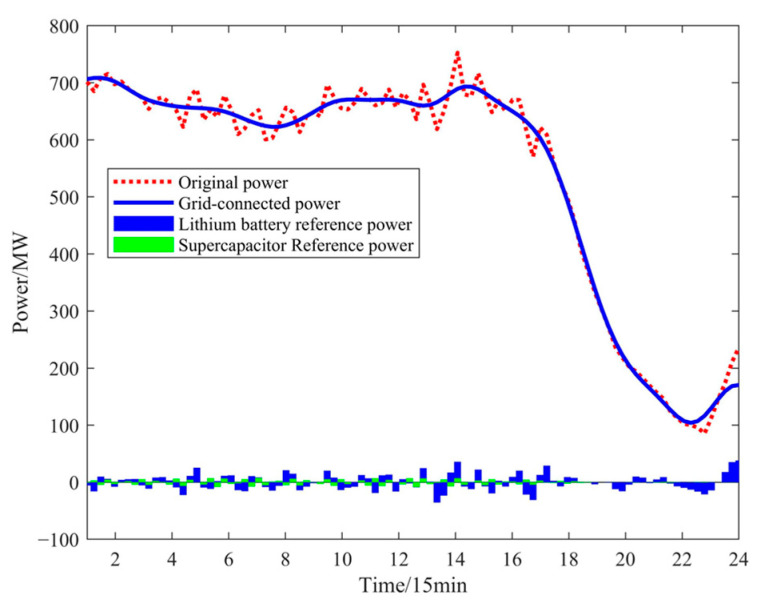
Wind-power reconstruction result.

When wind power is sufficient, its volatility is high, requiring wind-power smoothing. Firstly, the EEMD algorithm is used to decompose wind power into high-frequency and low-frequency signals. After low-frequency reconstruction, the low-frequency component is directly integrated into the grid, while the high-frequency component is smoothed by the hybrid energy storage system. The wind-power reconstruction results are shown in Figure 10. From the wind-power data chart, it can be seen that, as forecasting accuracy improves, the volatility and randomness of wind power become more pronounced, with wind-power fluctuations reaching 60.4 MW, which severely impacts the stability of the power system. To reduce this volatility, the original wind-power data are decomposed using the EEMD algorithm, followed by low-frequency reconstruction of the results. After reconstruction, the wind-power fluctuation amplitude is reduced to 39.35 MW, a 34.9% decrease compared to before processing. This makes the wind-power curve smoother and significantly improves the impact of wind-power fluctuations on the power system.

The optimized VMD parameters obtained through the NGO algorithm are shown in Table 3. In the sufficient wind-power scenario, the VMD parameters are optimized through NGO, resulting in *K* = 7 and *α* = 2813.

In Table 3, *D* is the population size; *I* is the number of iterations; *K* is the number of decomposed modes; *α* is the penalty factor; *init* is the initial modal frequency; *tol* is the tolerance value for the convergence criterion.

**Figure 11:**
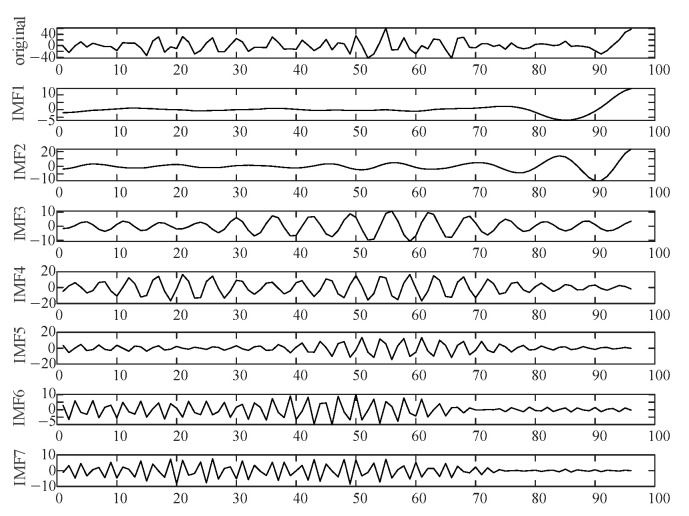
NGO-VMD decomposition frequency diagram.

**Figure 12:**
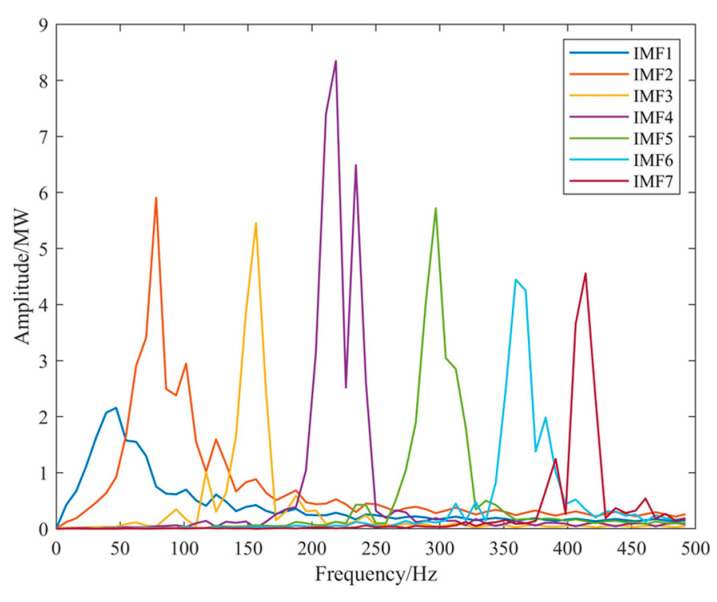
NGO-VMD marginal spectrum.

Using the above parameters for NGO-VMD decomposition, the resulting frequency decomposition and marginal spectrum are shown in Figure 11 and Figure 12. Since supercapacitor are a power-type energy storage with much higher costs than lithium battery, this paper primarily uses lithium battery storage, with a supercapacitor only handling a portion of the high-frequency components. Therefore, IMF 1-IMF 6 components are absorbed by the lithium battery, and the supercapacitor only handles the IMF 7 component.

### 4.3. Analysis of Intra-Day Optimization Scheduling Results

**Figure 13:**
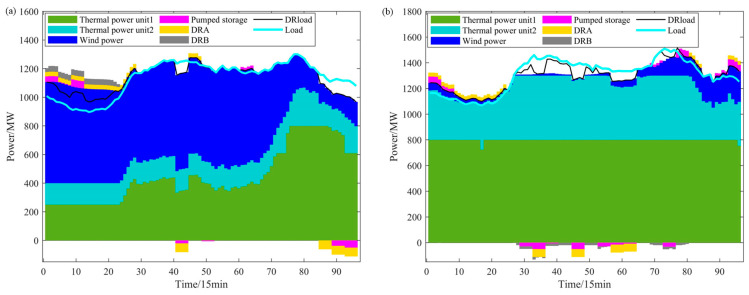
Intra-day scheduling results: (**a**) intra-day scheduling in the sufficient wind-power scenario; (**b**) intra-day scheduling in the insufficient wind-power scenario.

As shown in Figure 13a, the intra-day scheduling results under the sufficient wind-power scenario indicate that, in this scenario, the increase in electric arc furnace load occurs during the 1–24 period. During this period, after adjustments from the electrolytic aluminum, thermal-power units, and pumped storage, a large amount of wind power still cannot be absorbed. Apart from this, the wind power has already been fully consumed.

As shown in Figure 13b, the intra-day scheduling results for the insufficient wind-power scenario indicate that, under this scenario, the electric arc furnace load is primarily reduced, occurring mainly during the 29–56 and 69–81 time periods. During these periods, the wind-power output and thermal-power output are unable to meet the system load demand. Therefore, reducing the electric arc furnace load can help reduce load shedding costs. In addition, during the 68–72 time period, there is a contradiction between the pumped storage charging and the reduction in electric arc furnace load. This is because the intra-day forecast accuracy is higher, and, compared to the day-ahead forecast, the load and wind-power data have changed. The intra-day forecasted load is higher during this period, and the charging of pumped storage would cause the system load to be insufficient. Therefore, the reduction in electric arc furnace load is necessary to adjust and balance the system.

### 4.4. Analysis of Different Scheduling Strategies

To validate the effectiveness of the proposed strategy in this paper, comparative experiments were conducted using two other strategies:

Strategy 1: The strategy proposed in this paper.

Strategy 2: Demand response is achieved solely through the use of energy storage.

Strategy 3: The scheduling plan for adjustable resources is conducted only in the day-ahead stage.

Table 4 and Table 5 show the operating costs of different strategies in the sufficient wind-power and insufficient wind-power scenarios. In the sufficient wind-power scenario, strategy 1 effectively reduces wind curtailment costs by implementing an industrial load demand response, resulting in a total cost lower than that of strategy 2 and strategy 3. Strategy 2 utilizes only energy storage for demand response. Although it does not involve load scheduling or reserve costs, it fails to effectively reduce wind curtailment. As a result, its wind curtailment cost is 2.64 times that of strategy 1, making its total cost the highest. In strategy 3, the scheduling plan is determined day-ahead, with only the electrolytic aluminum load participating in demand response. Although its wind curtailment cost is 138,345 CNY lower than that of strategy 2, the overall cost remains higher than that of strategy 1. In the insufficient wind-power scenario, all wind power can be fully utilized, resulting in no wind curtailment costs. However, due to insufficient thermal- and wind-power generation, a large load-shedding cost is incurred. It can be observed that, in this scenario, the energy storage costs for all three strategies are identical, indicating that the energy storage adjustment capability has been fully utilized under wind-power deficiency. Therefore, strategy 1, which introduces electrolytic aluminum and electric arc furnace loads, has a significantly lower cost than strategy 2 and strategy 3. This demonstrates that, whether in the sufficient or insufficient wind-power scenarios, the proposed strategy effectively enhances system regulation capability and optimizes operational costs.

## 5. Conclusions

This paper presents a multi-time-scale scheduling model that integrates industrial loads and energy storage systems under high wind energy penetration. By introducing an energy storage system, it effectively facilitates the integration of a high percentage of wind power into the electricity grid, maximizing wind energy absorption through pumped storage and industrial loads. This scheduling model not only optimizes the utilization efficiency of wind power but also enhances the stability and flexibility of the electricity system. The main conclusions are as follows:

(1) Through analysis, this paper demonstrates the feasibility of electrolytic aluminum and electric arc furnace loads participating in demand response and provides a load model for load participation in scheduling. This offers a foundation for the scheduling of industrial loads in power systems, helping to enhance wind-power absorption capability.
(2) The intra-day smoothing strategy for wind-power fluctuations proposed in this paper effectively ensures the safety of integrating wind power into the electricity system at a finer time scale. By optimizing the scheduling of the hybrid energy storage system, this strategy successfully alleviates the impact of wind-power fluctuations on system stability.
(3) Through simulation examples, it has been verified that, in the sufficient wind-power scenario, the proposed scheduling strategy can significantly improve wind-power absorption capacity and reduce wind curtailment. In the insufficient wind-power scenario, it can effectively reduce load shedding, optimizing the overall operation of the power system.

## Figures and Tables

**Table 1:** Parameters of thermal-power units.

Unit	Pmax (MW)	Pmin (MW)	a (CNY/MW^2^)	b (CNY/MW)	c (CNY)	Ramp Rate (MW/h)	Start-Stop Costs (10^5^CNY)
1	800	250	0.00173	20.26	244.2	−250–250	9–4
2	500	150	0.00221	24.68	176.8	−150–150	9–3

**Table 2:** Energy storage device parameters.

Type	*P*_max_ (MW)	*η* (%)	SOE_min_ (%)	SOE_max_ (%)	*E*/MWh
Lithium battery	40	0.95	0.1	0.9	20
Supercapacitor	10	0.90	0.1	0.9	5
Pumped storage power station	50	0.85	0.1	0.9	250

**Table 3:** NGO-optimized VMD parameter settings.

*D*	*I*	*K*	*α*	*init*	*tol*
20	20	[4, 9]	[10, 5000]	1	1 × 10^−7^

**Table 4:** Operating costs of different strategies under sufficient wind-power scenario.

Type	Strategy 1	Strategy 2	Strategy 3
Thermal-power generation cost/CNY	335,188	340,410	335,839
Energy storage cost/CNY	73,743	87,848	73,743
Wind curtailment cost/CNY	169,391	448,528	310,183
Load dispatch cost/CNY	81,314	-	29,664
Load-shedding cost/CNY	0	0	0
Total cost/CNY	659,564	876,786	749,429

**Table 5:** Operating costs of different strategies under insufficient wind-power scenario.

Type	Strategy 1	Strategy 2	Strategy 3
Thermal-power generation cost/CNY	665,226	306,785	665,243
Energy storage cost/CNY	141,894	12,045	141,894
Wind curtailment cost/CNY	0	0	0
Load dispatch cost/CNY	63,913	-	29,664
Load-shedding cost/CNY	1,499,210	2,420,900	2,425,119
Total cost/CNY	2,370,241	3,226,106	2,961,920

## Data Availability

Data are contained within the article.
